# The promise and peril of database research: Common pitfalls and how to avoid them

**DOI:** 10.1016/j.xjon.2026.101618

**Published:** 2026-02-11

**Authors:** Nikki E. Rossetti, Nahom Seyoum, Varun Puri

**Affiliations:** Division of Cardiothoracic Surgery, Department of Surgery, Washington University School of Medicine, St Louis, Mo

**Keywords:** database research, observational studies, surgical outcomes, clinical registries, health services research, methodologic rigor

**Figure:**
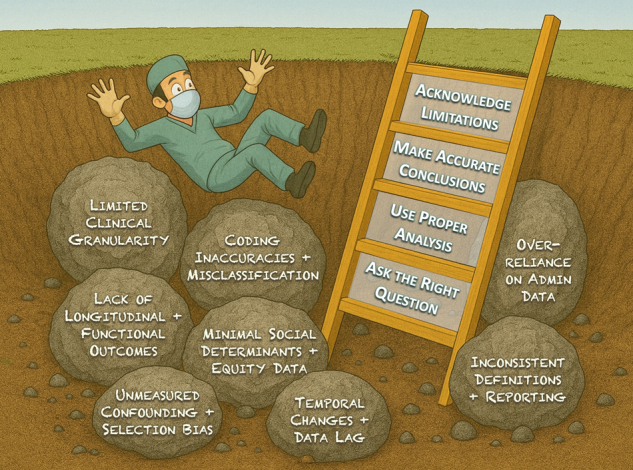
Climbing past investigative pitfalls: rigorous methods elevate database research.

**Central Message**

Thoughtful design, transparent methods, and clinical context transform imperfect large databases into vital tools that complement trials and advance evidence-based, patient-centered surgical care.

The emergence of electronic medical records, administrative claims data, and national registries has transformed how clinical outcomes are studied by providing accessible, high-value tools for examining real-world care, enabling analyses of rare events, and revealing patterns of care that are beyond the scope of prospective trials. These resources have informed guidelines, shaped policy, and supported quality improvement initiatives across multiple specialties.

Commonly used large national data registries include the Veterans Affairs Surgical Quality Improvement Program, the National Surgical Quality Improvement Program,^1^ the Surveillance, Epidemiology, and End Results-Medicare database,^2^ the National Cancer Database (NCDB), and the Society of Thoracic Surgeons (STS) Database.^3^ These programs have spurred a surge in retrospective cohort studies (Figure E1), reflecting both their scientific value and the academic drive for productivity, representing a “Field of Dreams” effect—if you build it [databases], they [researchers] will come.

However, the same attributes that make these datasets appealing (scale, speed, accessibility) also expose them to potential methodologic flaws. Without careful study design and thoughtful interpretation, database research is vulnerable to several investigative pitfalls that compromise study validity.^4^ Reporting frameworks such as Strengthening The Reporting of Observational studies in Epidemiology^5^ have improved observational research transparency, but they focus on study reporting rather than design. This review highlights both structural (nonmodifiable) and user-driven (modifiable) pitfalls in database research and provides pragmatic strategies to enhance rigor, interpretability, and clinical relevance.

## Nonmodifiable Pitfalls: Database Structural Limitations

Large-scale databases are inherently constrained by the way data are collected, coded, and maintained. Their structure and content impose some nonmodifiable limitations that investigators must recognize and accommodate during study design.

### Limited Granularity

What large clinical databases gain in scope, they often lose in depth; poor data granularity limits nuanced clinical assessment,^6^ robust risk adjustment, and between-group comparison. Key variables are frequently missing, including imaging findings, detailed cancer staging, frailty scores, molecular markers, smoking status, and severity of comorbid conditions.^3^^,^^6^ In previous work from our group, the absence of imaging data limited assessment of patient candidacy for sublobar lung cancer resection.^7^

Investigators should tailor research questions that can be answered with the available data and avoid inferences that depend on missing or unmeasured data. Strategies include the selection of datasets suited to the question, linking registries to complementary sources, and developing disease-focused data infrastructure. A study linking the STS General Thoracic Database to Medicare claims data demonstrated how combining datasets can enhance long-term outcomes data after lung cancer surgery.^8^ The new American Association for Thoracic Surgery Quality Gateway, which incorporates frailty data and patient-reported outcomes,^9^ exemplifies how modern registries can evolve. Continued progress will depend on shifting from specialty-to disease-focused registries that reflect the complexity of contemporary care.^10^

### Unmeasured Confounding and Selection Bias

Unmeasured confounders, variables associated with both exposure and outcome that are not captured in the dataset, can distort apparent causal associations. Large registry studies are especially vulnerable because baseline differences between cohorts are often incompletely captured. This is evident in the NCDB, where several important cancer-related variables are missing, including smoking status, frailty, or imaging findings.^11^

Fragmented data systems further contribute to selection bias by producing study populations that differ from the broader target population. For example, coronary artery bypass graft procedures are recorded in the STS Adult Cardiac Surgery Database, whereas percutaneous coronary interventions appear in the National Cardiovascular Data Registry, despite both addressing the same underlying condition (coronary artery disease). Dataset linkage through collaborative models such as the STS-American College of Cardiology Transcatheter Valve Therapy Registry demonstrate how cross-specialty coordination can reduce data fragmentation and strengthen analytic validity by capturing more decision-related.

Analytic tools, including the E-value and bounding factor B, can quantify the robustness of causal inferences to unmeasured confounding,^12^ but no statistical method substitutes careful design. Transparent acknowledgement of limitations remains central to credible observational research.^5^

### Coding Inaccuracies, Misclassification, and Data Evolution

In administrative databases, reliance on *International Classification of Diseases* (ICD) and Current Procedural Terminology codes introduces risk of misclassification, stemming from billing priorities, ambiguous definitions, and inconsistent documentation.^13^

Coding inaccuracies are common in cancer-related variables such as tumor histology, staging, treatment, and recurrence, resulting in substantial data missingness (up to 67.8% for some variables in the NCDB)^14^ and variation in basic outcomes definitions such as “mortality,” which may refer to in-hospital, 30-day, or 90-day death. Our group has demonstrated that integrating multiple Veterans Affairs data sources can effectively reconcile missing or inconsistent outcomes and improve data completeness.^15^

Temporal changes further compound these challenges. Coding transitions (such as the shift from ICD Ninth Revision to the ICD Tenth Revision), evolving variable definitions, data-release delays, and evolving treatment paradigms over time complicate longitudinal analyses and dataset linkages.^16^ When databases are merged across eras or sources, investigators risk introducing systematic error if these temporal dynamics are not explicitly addressed.

To enhance reliability, investigators should avoid single-code definitions, use composite algorithms, validate key variables through manual chart review when feasible, and transparently describe coding limitations and temporal assumptions.

### Inability to Capture Long-Term or Functional Outcomes

Most registries emphasize short-term outcomes and, consequently, underrepresent end points that matter most to patients, including recurrence-free survival, functional status, and quality of life.^10^ This represents one of the most consequential constraints of database research, because it relies on substitution of short-term proxies for long-term outcomes.

Linking registries with claims data or institutional electronic medical records can help bridge this gap.^4^ The STS has begun incorporating long-term follow-up and patient-reported outcomes,^3^ but broader adoption is needed to ensure the data reflect patient priorities.^10^ When long-term or functional outcomes are unavailable, investigators should acknowledge this limitation and avoid extrapolating short-term findings beyond what the data support.

### Missing Context: Social and Equity-Relevant Data

Large datasets often lack the social and structural context needed to interpret outcomes disparities. Variables related to social determinants of health (eg, race, income, geography, and support systems) are frequently missing or oversimplified, limiting equity-focused analyses.^17^ Although these limitations may not be fully correctable within existing datasets, investigators should interpret findings cautiously and advocate for improved data capture in future registry iterations.

## Modifiable Pitfalls: User-driven Design and Analysis Errors

Although structural limitations are inherent to large datasets, the most consequential shortcomings of database research arise from investigator decisions. These modifiable pitfalls, which include poorly framed questions, analytic missteps, and overinterpretation, can be avoided through thoughtful study design, multidisciplinary input, and methodologic rigor. The following sections highlight common errors and practical strategies to avoid them.

### Asking Unanswerable, Inappropriate, or Meaningless Questions

An easily avoidable user error is posing questions that the data cannot answer or that lack clinical relevance.^11^ When exposure groups do not plausibly overlap in treatment eligibility or baseline risk, no amount of statistical adjustment can recover valid comparative inference, and such questions are fundamentally unanswerable regardless of database size.

Related examples of poorly framed questions include several studies^18^, ^19^, ^20^ that examined seasonal variation in clinical outcomes such cancer risk, wound healing, or surgical complications. Although these studies achieved statistical significance, they consumed academic resources and journal space with questionable clinical impact. To promote meaningful inquiry, institutions should consider internal vetting processes that include multidisciplinary teams of clinical and data experts. External review, such as the process once required for NCDB data access, can further strengthen methodologic rigor and ensure the selected database is appropriate for the study question.

### Overinterpretation or Misrepresentation of Findings

Large datasets confer immense statistical power, allowing even trivial effects to reach statistical significance and tempting investigators to overstate findings.^11^ In a review of >300 studies published in leading surgical journals, only 8.5% specified clinically meaningful effects.^21^ Overinterpretation of statistically significant but trivial findings can mislead readers and distort policy or guideline development. Investigators should emphasize effect sizes, CIs, and absolute differences and clearly distinguish statistical significance from clinical importance.

### Methodologic Hazards

#### Selection of wrong database (right question, wrong dataset)

Another common pitfall occurs when investigators attempt to answer complex questions using datasets that lack sufficient clinical detail. For example, evaluating outcomes such as recurrence or functional recovery in databases without imaging data or long-term patient-reported outcomes weakens internal validity and invites speculative conclusions. In addition, the NCDB may be unsuitable for assessing comorbidities because its data lack limited accuracy and depth, whereas VA datasets provide more granular and validated comorbidity information. Early collaboration with analytic methodologists and cross-disciplinary clinical experts can help align research aims with dataset capabilities and ensure analyses are both credible and clinically meaningful.

#### Misapplication of statistical methods (right question and database, wrong analysis)

Even well-conceived studies can fail when analytic methods do not align with the chosen question or dataset. Common mistakes include applying parametric tests to categorical variables, ignoring time-to-event outcomes, or omitting key covariates in observational analyses.^11^^,^^22^ In large samples, these errors can yield results that appear statistically precise but are clinically or methodologically invalid. Collaboration with statisticians who understand both the dataset and the clinical context is essential. The chosen analytic approach must fit the study design and data structure to ensure valid and interpretable conclusions.

#### Failure to address data complexity and biases

Immortal time bias occurs when exposure is defined using information available only after follow-up begins, creating a time window during which outcomes cannot occur. For example, when patients are classified as “surgical” only after surviving the perioperative period.^23^ This artifact can inflate survival estimates.

Clustering is another pervasive issue. Outcomes of patients treated at the same hospital or by the same physician are often correlated, even after adjusting for patient characteristics. Ignoring this violates the assumption of independent observations and underestimates variance. Multilevel or mixed-effects models that incorporate provider- or facility-level random effects are appropriate remedies.^23^

Missing data also threaten validity if not addressed systematically. Investigators should determine whether missingness is completely random, at random, or not at random. Complete-case analysis is valid only under missingness is completely random. Alternatives such as multiple imputation, maximum likelihood estimation, or inverse probability weighting should be chosen based on the mechanism of missingness.^23^ Sensitivity analyses can further assess the potential impact of missing data.

### Why Database Research Still Matters

Despite limitations, large clinical databases remain indispensable to health services and outcomes research. They provide insights that randomized trials often cannot, particularly where prospective studies are infeasible, prohibitively expensive, or ethically challenging.^24^ In addition, they enable benchmarking and quality improvement initiatives across diverse health care settings, exemplified by the American Association for Thoracic Surgery Quality Gateway, which leverages registry data to monitor outcomes and inform thoracic surgical practice.^9^ The power of database studies can shape policy development and innovation. A notable example is a retrospective cohort study that has helped refine national lung cancer screening guidelines to include more high-risk patients.^25^

Artificial intelligence, machine learning, and natural language processing are transforming database research through automation and prediction modeling. However, these innovations bring new challenges. Advances in quantum computing may soon facilitate unprecedented analytic efficiency, which risks a surge of studies lacking clinical significance and/or analytic rigor. As these technologies emerge, investigators must pair innovation with discipline, ensuring that artificial intelligence enhances, rather than replaces, thoughtful design, transparency, and clinical insight.

New analytic tools will continue to reshape how surgical research is conducted, but the present and future of rigorous database science depend on researchers’ ability to recognize the common pitfalls outlined in this review. The ladder out of the metaphorical pit has 4 rungs: (1) asking clinically insightful study questions, (2) using the proper dataset and analytic approach, (3) drawing accurate conclusions, and (4) transparently acknowledging limitations. When applied thoughtfully, these methods help leverage the scalability and inclusivity of databases to capture the complexity of real-world care and complement randomized trials to advance evidence-based, equitable surgical practice.

### Ethics Statement

This article is an expert opinion and methodologic review and did not involve human participants, patient-level data, or identifiable private information. Therefore, institutional review board approval was not required, and informed written consent was not applicable.

## Conflict of Interest Statement

The authors reported no conflicts of interest.

The *Journal* policy requires editors and reviewers to disclose conflicts of interest and to decline handling or reviewing manuscripts for which they may have a conflict of interest. The editors and reviewers of this article have no conflicts of interest.

## Figures and Tables

**Figure E1:**
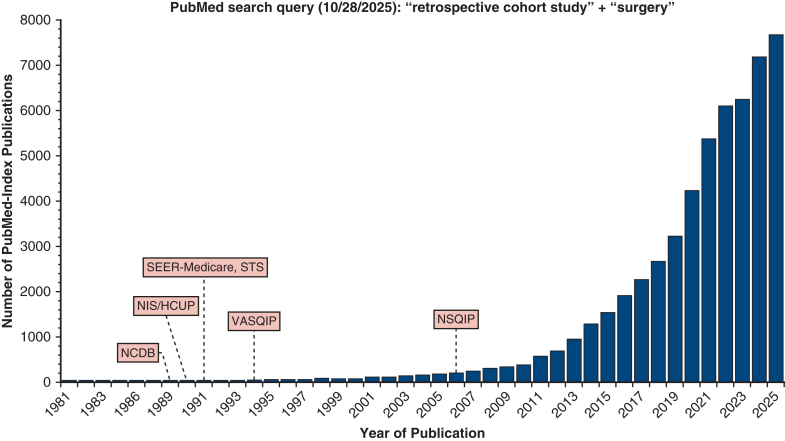
Growth in PubMed-indexed surgical retrospective cohort studies over time. The release of key national databases that facilitated this expansion are highlighted. *SEER-Medicare*, Surveillance, Epidemiology, and End Results-Medicare; *NIS*, National Inpatient Sample; *HCUP*, Healthcare Cost and Utilization Project; *NCDB*, National Cancer Database; *VASQIP*, Veterans Affairs Surgical Quality Improvement Program; *NSQIP*, National Surgical Quality Improvement Program.
